# Effects of Lifestyle Modifications on Prediabetic Patients: A Quasi-Experimental Trial

**DOI:** 10.7759/cureus.78062

**Published:** 2025-01-27

**Authors:** Dileep Kumar, Shehzad Hussain, Binjo Jose Vazhappilly, Sadia Akbar, Ahmed Fathy Maarouf

**Affiliations:** 1 Department of Cardiology, Phoenix Hospital, Abu Dhabi, ARE; 2 Department of Emergency Medicine, Phoenix Hospital, Abu Dhabi, ARE; 3 Department of Internal Medicine, Phoenix Hospital, Abu Dhabi, ARE

**Keywords:** glycemic control, glycosylated hemoglobin (hba1c), lifestyle interventions, prediabetes, prevention of diabetes

## Abstract

Introduction: Prediabetes is characterized by impaired fasting glucose or impaired glucose tolerance (IGT), which can lead to cardiovascular complications. This study aims to determine the effects of lifestyle modification on glycemic outcomes in prediabetic individuals.

Methods: This quasi-experimental trial was conducted at the Department of Adult Cardiology, Phoenix Hospital, Abu Dhabi. Participants aged ≥18 years, of either gender, diagnosed with prediabetes as defined by hemoglobin A1c (HbA1c) levels between 5.7% and 6.4% were included in the study. Participants underwent a three-month lifestyle modification, adapted from cardiac rehabilitation principles, which included glycemic control, nutritional counseling, physical activity counseling, and exercise training. The primary outcome was the change in HbA1c levels following the intervention. Data analysis was performed using RStudio (Posit Software, Boston, MA).

Results: A total number of 101 participants were enrolled, and 96 people completed the study. The average age of the participants was 44.80 ± 8.27 years. Most individuals were males, 85 (88.5%), and 11 females accounted for 11.5%. Postintervention, 25 participants (26%) reverted to normoglycemia, while 67 (69.8%) remained prediabetic, and 4 (4.2%) progressed to diabetes. Significant reductions in HbA1c levels were observed (p < 0.05). Subgroup analysis revealed no significant differences in outcomes based on age, gender, or clinical characteristics. The program demonstrated strong adherence, with a retention rate of 95%.

Conclusion: Lifestyle modification effectively improved glycemic control and reduced the progression of diabetes in prediabetic individuals. The findings support the integration of lifestyle interventions for managing prediabetes.

## Introduction

Prediabetes is characterized by impaired fasting glucose (IFG) or impaired glucose tolerance (IGT). It has emerged as a critical precursor to type 2 diabetes mellitus and the development of cardiovascular complications, including myocardial infarction, heart failure, and atherosclerosis [1-3]. Globally, the prevalence of prediabetes continues to rise, posing a substantial public health challenge [2,3]. Additionally, it is estimated that the prevalence of IGT is approximately 7.5% in both men and women worldwide [4]. Projections suggest that by 2045, the prevalence of IFG will rise by 6.5% to affect 414 million individuals, while IGT prevalence is expected to increase by 10%, impacting 638 million people globally [2]. Low- and middle-income countries (LMICs) are disproportionately affected by prediabetes (72%), where resources for prevention and management are often limited [4]. Regional variations in prevalence further highlight disparities, with North America and the Caribbean showing the highest rates of IGT (14%) compared with Europe, which reports significantly lower rates (5.1%) [4].

Lifestyle interventions encompassing dietary modifications, physical activity, and behavioral changes have been widely recognized as the cornerstone for preventing the progression of prediabetes to diabetes [5]. While traditional cardiac rehabilitation programs focus on improving cardiovascular health under medical supervision, their principles can be adapted for noncardiac populations [6]. Lifestyle rehabilitation modifies these principles for prediabetic patients, integrating structured dietary guidance, exercise programs, and psychosocial support to mitigate the risks associated with prediabetes [6-8].

Evidence from several pivotal studies has established that lifestyle interventions, encompassing dietary changes, physical activity, and behavioral modifications, can significantly mitigate the progression to diabetes, with risk reduction ranging between 30% and 60% [9-11]. However, the persistence of prediabetes, compared to its reversal to normal glucose regulation, remains a significant challenge, particularly among individuals undergoing lifestyle modifications [11,12].

Despite advances in diabetes prevention strategies, there is limited clarity regarding the onset and duration of prediabetes in individuals [5,7,13]. Existing data often focus on the time from initial diagnosis of prediabetes to intervention rather than capturing the entire trajectory of the condition [5]. This gap emphasizes the need for further studies focusing on structured lifestyle interventions in populations with prediabetes. Hence, this study aims to evaluate the effectiveness of lifestyle modification in prediabetic patients. By tailoring interventions to the needs of this population, the study would provide actionable insights into reducing diabetes progression and improving overall metabolic health. This approach can potentially address the growing burden of prediabetes and offer a sustainable model for global implementation.

## Materials and methods

This quasi-experimental trial was conducted at the Department of Adult Cardiology, Phoenix Hospital, Abu Dhabi, between March 21, 2024, and October 30, 2024. The sample size was determined using the WHO sample size calculator, based on an estimated 39% postintervention normalization rate of glucose levels [14] with a 10% margin of error and a 5% significance level. To account for potential dropouts, the sample size was inflated by 10%, resulting in a total of 101 participants. Eligibility criteria included individuals aged 18 years or older, of either gender, diagnosed with prediabetes as defined by hemoglobin A1c (HbA1c) levels between 5.7% and 6.4%, and willing to participate for three months. Exclusion criteria were unstable angina, complex ventricular arrhythmias, recent thrombophlebitis with or without pulmonary embolism, acute decompensated congestive heart failure, uncontrolled inflammatory or infectious conditions, intracavitary thrombus, severe or symptomatic aortic stenosis, severe obstructive cardiomyopathies, severe pulmonary hypertension (right ventricular systolic pressure >60 mm Hg), and musculoskeletal disorders impeding participation in exercise. Participants were recruited using a nonprobability consecutive sampling technique.

Participants were enrolled after obtaining ethical approval (Research Ethics Committee approval no. MF2482-2024-1) and written informed consent. Baseline demographic and clinical data were collected for all participants. Patients were managed according to standardized institutional protocols and evidence-based guidelines.

The intervention consisted of a three-month lifestyle modification adapted from cardiac rehabilitation principles. It included nutritional counseling, physical activity counseling, and exercise training.

All of these participants were advised to follow a simple plan for three months, including cutting down on or stopping sugar and sugary items, decreasing carbohydrate content, especially rice, eating fruits and vegetables at least once instead of lunch or dinner, not drinking soft drinks and packaged juices, and stopping eating all kinds of meat except grilled chicken and grilled fish. They were also advised to engage in some physical activity, such as 40 minutes of brisk walking, jogging, or playing any active sports.

The primary outcome was the change in HbA1c levels following the three-month intervention. Data were collected systematically using a structured questionnaire.

Data analysis was performed using RStudio (Posit Software, Boston, MA). Continuous variables, such as age, HbA1c, low-density lipoprotein, and triglycerides, were summarized as mean ± standard deviation or median and interquartile range based on the data distribution. Categorical variables, including gender, cardiovascular disease (CVD), hypertension, dyslipidemia, and prediabetic status postintervention, were presented as frequencies and percentages. Wilcoxon signed-rank tests were applied to compare pre- and postintervention HbA1c levels. Stratification was conducted to assess the influence of age, gender, CVD, hypertension, and dyslipidemia on the outcomes, with poststratification comparisons performed using Fisher’s exact test. A p value of ≤0.05 was considered statistically significant.

## Results

The study began with an enrollment of 101 participants; however, five individuals did not complete the follow-up, resulting in a final analysis of 96 participants. The average age of the participants was 44.80 ± 8.27 years. The majority were male (88.5%), with females accounting for 11.5%. Additionally, 39.6% of the participants had hypertension, 6.3% had CVD, and 44.8% were diagnosed with dyslipidemia (Table 1).

**Table 1:** Descriptive analysis of baseline characteristics of the participants CVD: cardiovascular disease; LDL: low-density lipoprotein; TG: triglycerides

Characteristics	Statistics
Age (years)	44.80 ± 8.27
Gender	
Female	11 (11.5)
Male	85 (88.5)
Hypertension	
Yes	38 (39.6)
No	58 (60.4)
CVD	
Yes	6 (6.3)
No	90 (93.8)
Dyslipidemia	
Yes	43 (44.8)
No	53 (55.2)
LDL (mg/dL)	128.75 ± 36.236
TG (mg/dL)	129 (101-214)

**Figure 1:**
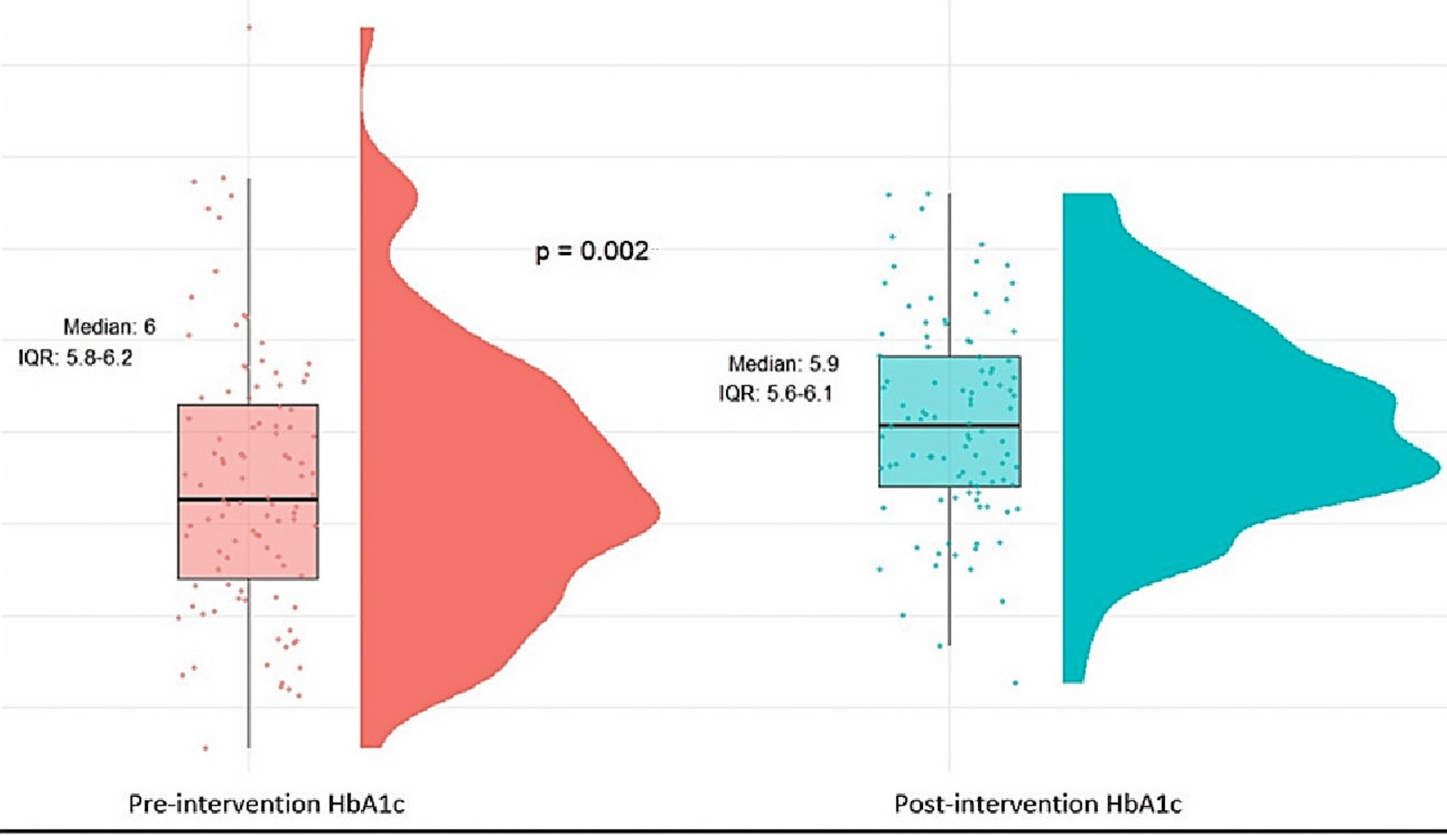
Comparison of pre- and postintervention HbA1C levels IQR: interquartile range; HbA1C: hemoglobin A1c

Before the intervention, all participants were categorized as prediabetic based on HbA1c levels ranging from 5.7% to 6.4%. Following the intervention, a significant decrease in HbA1c levels was recorded, highlighting the effectiveness of the lifestyle modification (p = 0.002) (Figure 1).

About 67 (69.8%) participants remained in the prediabetic range, while 25 (26%) achieved normal glycemia. Additionally, four of the participants (4.2%) progressed to diabetes. When stratified by age, gender, hypertension, dyslipidemia, and CVD, no significant differences were observed in postintervention outcomes. Participants aged ≤45 and >45 years exhibited similar glycemic responses. Similarly, no significant variations were found between genders or based on the presence of hypertension or dyslipidemia. All participants with CVD remained in the prediabetic range after the intervention (Table 2).

**Table 2:** Postintervention glycemic outcomes stratified by age, gender, hypertension, dyslipidemia, and CVD Fisher's exact test was applied CVD: cardiovascular disease; HTN: hypertension

Variables	Normal	Prediabetes	Diabetes	p value
Age groups				
≤45 years	12	32	2	0.57
>45 years	13	35	2	
Gender				
Male	22	59	4	0.47
Female	3	8	0	
CVD				
Yes	0	6	0	0.25
No	25	61	4	
HTN				
Yes	11	25	2	0.468
No	14	42	2	
Dyslipidemia				
Yes	9	33	1	0.357
No	16	34	3	

## Discussion

The current study evaluated the effects of lifestyle modification on glycemic outcomes in prediabetic individuals. Significant improvements were observed, with 25.7% of participants reverting to normoglycemia, 69.7% remaining in the prediabetic range, and 4.6% progressing to diabetes. The program, which included dietary counseling, physical activity, and psychosocial support, demonstrated a clear impact on reducing HbA1c levels. These findings are consistent with other studies supporting the efficacy of lifestyle interventions in managing prediabetes. For instance, the Diabetes Prevention Program (DPP) reported a 58% reduction in diabetes incidence with intensive lifestyle interventions (p < 0.001), similar to the Finnish Diabetes Prevention Study, which showed a 58% relative risk reduction over 3.2 years (p < 0.001) [15]. The Indian Diabetes Prevention Program demonstrated a 28.5% reduction in diabetes progression over 30 months (p = 0.018), closely aligning with the glycemic improvements observed in this study [9]. Similarly, the Da Qing IGT and Diabetes Study reported a 43% reduction in diabetes incidence over six years (p < 0.05) through combined dietary and exercise interventions [10]. However, the short duration of the present study (three months) limits direct comparisons with these long-term trials.

Further comparisons with global data highlight the robustness of lifestyle interventions across diverse populations. The Study on Lifestyle Intervention and Impaired Glucose Tolerance Maastricht conducted in The Netherlands achieved a 47% reduction in diabetes risk (p = 0.035), while the ACT NOW study in the United States reported a 72% reduction in diabetes progression among participants with IGT (p < 0.001) [16,17]. Both studies validate the potential of lifestyle modifications to improve glycemic control, though differences in intervention durations and sample characteristics may explain slight variations in outcomes. Notably, a meta-analysis by Kerrison et al. reported a pooled diabetes risk reduction of 50% (p < 0.05), further corroborating the findings of this study [18]. Another meta-analysis by An et al. revealed a 17% reduction in all-cause mortality (RR, 0.83; 95% CI, 0.70-0.98) and a 38% reduction in retinopathy incidence (RR, 0.62; 95% CI, 0.70-0.98) with early and effective interventions. These outcomes emphasize the broader health benefits of lifestyle interventions, extending beyond glycemic control to include reductions in mortality and microvascular complications. Additionally, the patients with a 10-year cardiovascular risk >10% experienced greater benefits (p = 0.01), suggesting the potential for targeting high-risk groups for enhanced outcomes [19].

The research conducted by Kumari et al. reinforces the positive outcomes of the lifestyle intervention holistic (LIH) model for type 2 diabetes patients [20]. Their findings revealed notable decreases in fasting blood sugar (0.26 mg/dL), postprandial glucose (70.16 mg/dL), and HbA1c levels (2.82%). Additionally, the study indicated reductions in medication use (p < 0.004), hospitalization (p < 0.011), and surgical costs (p < 0.0005) among participants in the Lifestyle Intervention Counseling group. Improvements in quality of life and adherence to the LIH model were also noted. Although focused on a diabetic population, these results highlight the broader applicability of lifestyle-based counseling in reducing glycemic levels and healthcare costs, which could translate effectively to prediabetic populations.

The effectiveness of lifestyle interventions is further emphasized by Kumar et al., who evaluated nonpharmacological strategies in rural Indian communities [21]. Their study demonstrated significant improvements in modifiable cardiometabolic risk factors, including diastolic blood pressure (p = 0.017), systolic blood pressure (p = 0.008), waist circumference (p = 0.001), and waist-to-hip ratio (p = 0.001). Similar to our findings, Kumar et al. showcased the potential of integrating diverse lifestyle components, such as yoga and dietary counseling, to achieve broader health benefits.

The long-term effects of lifestyle interventions on mortality remain inconclusive. Evidence from the extended follow-up of the DPP and the DPP outcomes study revealed that although both lifestyle modification and metformin effectively delayed the progression to diabetes, they did not result in significant reductions in all-cause, cancer-related, or cardiovascular mortality over a median follow-up of 21 years. The hazard ratios for all-cause mortality were reported as 1.02 (95% CI, 0.81-1.28) for lifestyle modification and 0.99 (95% CI, 0.79-1.25) for metformin [22]. These findings imply that while lifestyle interventions address diabetes progression, their impact on mortality might be limited by factors unrelated to glycemic control.

Conversely, a systematic review and meta-analysis conducted by An et al. demonstrated a 17% reduction in all-cause mortality (RR, 0.83; 95% CI, 0.70-0.98) and a 38% reduction in retinopathy incidence (RR, 0.62; 95% CI, 0.70-0.98) in prediabetic patients without a history of CVD [19]. These findings underscore the importance of intervention timing, patient-specific risk factors, and sufficient follow-up duration in achieving optimal long-term outcomes. Notably, younger individuals, women, and patients with a 10-year cardiovascular risk exceeding 10% derived the greatest mortality benefits [19].

Adding further evidence, Dixit et al. demonstrated complete diabetes reversal in patients with HbA1c levels of up to 15% through targeted lifestyle interventions focused on weight reduction and enhanced insulin sensitivity. The cost-effectiveness of such interventions, particularly in resource-limited settings like LMICs like India, highlights their scalability and practical implementation potential [23,24]. Collectively, these studies reinforce the outcomes of this research, affirming the vital role of lifestyle interventions in prediabetes management while recognizing the need for tailored approaches to maximize long-term benefits.

Interestingly, our stratification analysis revealed no significant differences in outcomes across subgroups such as age, gender, hypertension, dyslipidemia, or CVD. This finding contrasts with certain international studies, such as Saito et al., which observed age and BMI as factors influencing glycemic responses [25]. However, the observation that participants with CVD remained in the prediabetic range aligns with findings from Kerrison et al., An et al., Kumari et al., and Kumar et al., emphasizing the need for targeted strategies for individuals with elevated cardiovascular and metabolic risk [18-21]. Future studies should incorporate cardiovascular risk assessments and explore additional interventions for high-risk populations.

The study has several strengths that enhance its validity and relevance. The use of a comprehensive lifestyle rehabilitation program addressing diet, exercise, and psychosocial factors ensured a holistic approach to prediabetes management. Conducting the intervention in a real-world clinical setting adds practical value, reflecting the feasibility of implementing such programs in routine healthcare practice. Robust statistical analyses, including stratification and the use of appropriate nonparametric tests, further strengthen the credibility of the results. The high retention rate (95%) is another significant strength, indicating strong participant engagement and adherence to the program. However, several limitations warrant attention in future research. The intervention’s three-month duration offers only a short-term perspective, limiting insights into sustained glycemic control and long-term diabetes prevention. The absence of a control group restricts the ability to directly compare outcomes with standard care or placebo, which impacts the generalizability of the findings. Furthermore, the single-center study design and relatively small sample size of 96 participants may not adequately reflect diverse populations and the short duration of the study. The reliance on self-reported adherence data introduces the potential for bias, and the lack of objective monitoring tools, such as wearable activity trackers, could affect the accuracy of compliance assessments. Future studies should aim to incorporate extended follow-up periods, integrate cardiovascular risk assessments, and explore scalable, cost-effective strategies for implementing lifestyle interventions. This approach will ensure that such programs can be tailored to diverse populations and settings, particularly in resource-limited environments, while providing a deeper understanding of their long-term impact.

## Conclusions

The lifestyle modification effectively improved glycemic control and reduced the progression of diabetes in prediabetic individuals. The findings support the integration of lifestyle interventions for managing prediabetes. Future research should focus on long-term sustainability and cost-effective scaling in diverse populations.
